# Computer-Aided excision of Parapharyngeal Space Pleomorphic Adenoma by Transcervical Approach. Case report

**DOI:** 10.4317/jced.62547

**Published:** 2025-03-01

**Authors:** Clara López-Martínez, Íñigo Aragón-Niño, Alba García-López-Chicharro, Marta María Pampín-Martínez, José Luis Cebrián-Carretero

**Affiliations:** 1 Oral and Maxillofacial Surgery Department, University Hospital La Paz, Madrid, Spain; 2 Oral and Maxillofacial - Head & Neck Oncology Department, Ninth People’s Hospital. Shanghai, China

## Abstract

**Background:**

The parapharyngeal space, situated in the neck’s lateral part, poses challenges for surgical intervention due to its complex anatomy. Tumors in this space are rare, accounting for 0.5-1% of head and neck tumors, often remaining asymptomatic until reaching substantial sizes.

**Material and Methods:**

This article outlines the surgical management of such lesions using navigation software, illustrated through a clinical case. A 38-year-old male presented with a mass at the right mandibular angle. Preoperative planning with Brainlab iPlan CMF® software aided in understanding the tumor’s relationship with adjacent structures. A transcervical approach facilitated tumor excision without damaging vital anatomical structures. Histopathology confirmed pleomorphic adenoma.

**Results:**

The paper underscores the rarity of parapharyngeal tumors, the importance of individualized surgical strategies, and the utility of 3D technology in preoperative planning for enhanced anatomical understanding.

**Conclusions:**

The cervical approach remains a satisfactory choice for benign lesions in this challenging space.

** Key words:**Parapharyngeal space, 3D technology, pleomorphic adenoma, transcervical approach, virtual planning.

## Introduction

The parapharyngeal space is a complex and deep region located in the lateral part of the neck. It takes the shape of an inverted triangle, being its upper limit the base of the skull, and its lower limit the hyoid bone. This space is divided into an anterior and a posterior compartment by the fascia inserted between the styloid process and the tensor veli palatini muscle. In the anterior compartment fat and salivary tissue are found. Meanwhile, the posterior compartment houses the carotid artery and the internal jugular vein, as well as cranial nerves IX, X, and XII (1). Tumors located in this region are rare, accounting for 0.5-1% of all head and neck tumors, with 80% being benign (2). Given their deep location, they often have a relatively long progression before symptoms arise, reaching large volumes at the time of diagnosis. They classically present as a medial displacement of the lateral wall of the oropharynx, amygdala, and/or soft palate or a mass at the level of the mandibular angle causing pain, dysphagia, or even involvement of cranial nerves (VII, IX, X, XI or XII) (3).

The primary tumors appearing in this space have varied histology: 35-45% of salivary origin; 35-41% of neurogenic origin; and others, such as hemangiomas, meningiomas, or lipomas, become extremely rare. Pleomorphic adenomas are the most frequent histological type in the anterior compartment and schwannomas are the most frequent lesions in the posterior compartment (3).

The treatment of benign tumors of the salivary gland in this space is mainly surgical. Surgical strategies to approach this space represent a challenge due to the depth of the lesions, the complex anatomy of this space, and the structures involved in this region (4).

In this context, this paper aims to present our experience in the surgical management of these lesions planned through navigation software. This approach is illustrated through a clinical case treated by the Oral and Maxillofacial Surgery Department of this center.

## Case Report

A 38-year-old male was evaluated at an external consultation for a non-painful mass at the right mandibular angle associated with dysphagia of unknown duration. Intraoral examination revealed a mass displacing medially the lateral wall of the oropharynx. On cervical examination, a mass was observed under the angle of the mandible. The mass was firm, non-compressible, non-pulsatile, and non-painful. For diagnosis and characterization of the lesion, a contrast-enhanced computed tomography scan of the neck was performed. It showed a lesion in the deep portion of the right parotid gland of 32 x 60 x 67 mm, which displaced medially the lateral pharyngeal wall, displaced anteriorly the pterygoid muscles, and was in intimate contact with the carotid vascular sheath. In addition, a histological study was performed with a fine needle puncture (FNA) biopsy and the result was suggestive of tissue compatible with pleomorphic adenoma.

**Figure 1:**
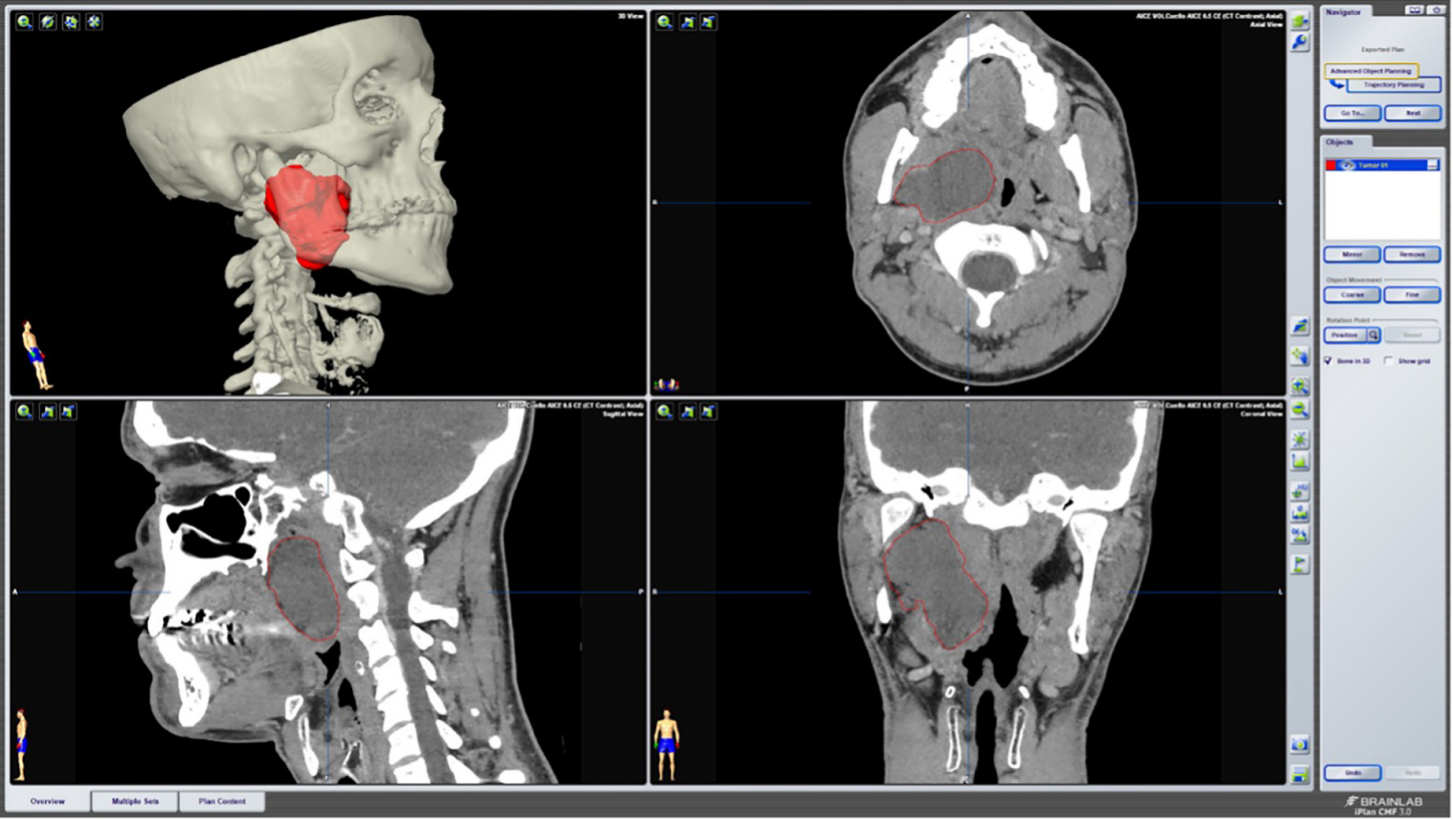
Utilization of Brainlab iPlan CMF software for pre-surgical planning, facilitating precise segmentation of the lesion.

After a complete evaluation of the lesion and diagnostic orientation, surgical excision of the tumor was planned. The Brainlab iPlan CMF® software (Brainlab AG, Munich, Germany) was used to carry out the segmentation of the lesion using the “Smart brush” tool. The pre-surgical planning allowed for a better understanding of the patient’s anatomy and the relationship between the tumor and other relevant anatomical structures such as the carotid vascular sheath (Fig. 1). During surgery, images and the preoperative plan were available.

**Figure 2:**
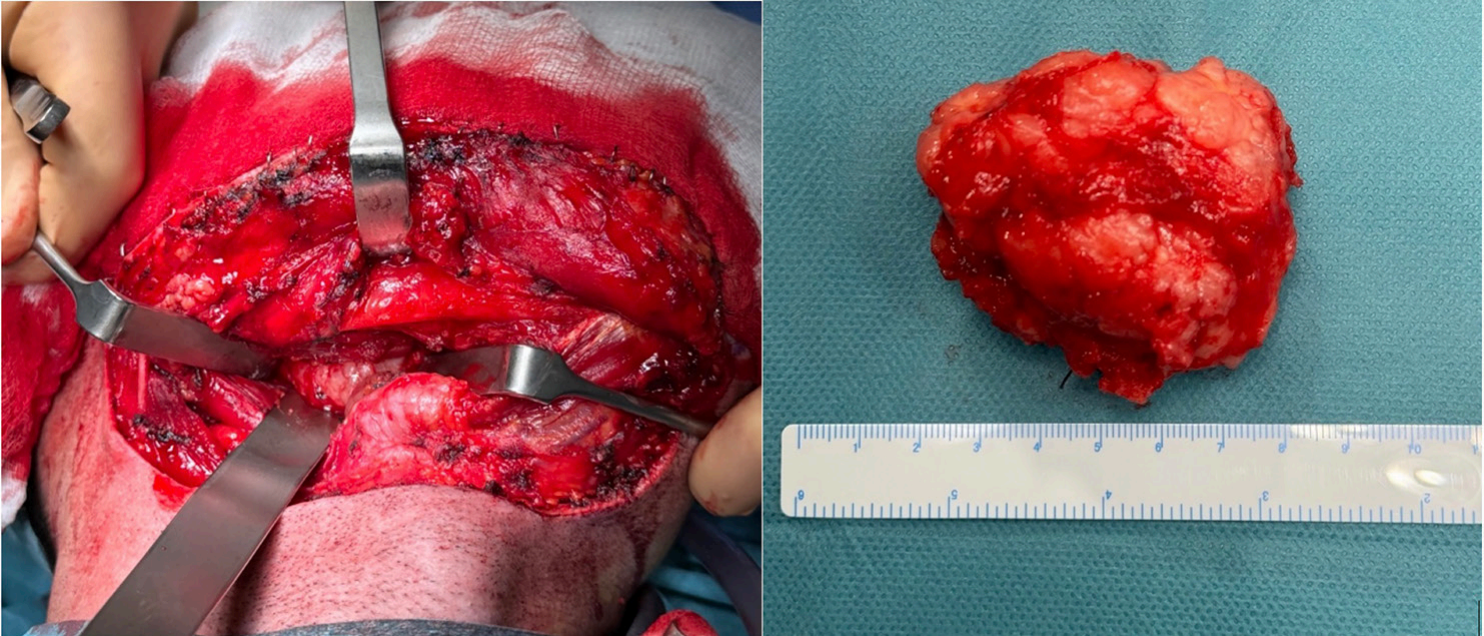
Identification of the mass followed by a careful blunt dissection, respecting the lesion’s capsule, and successful excision of the tumor.

**Figure 3:**
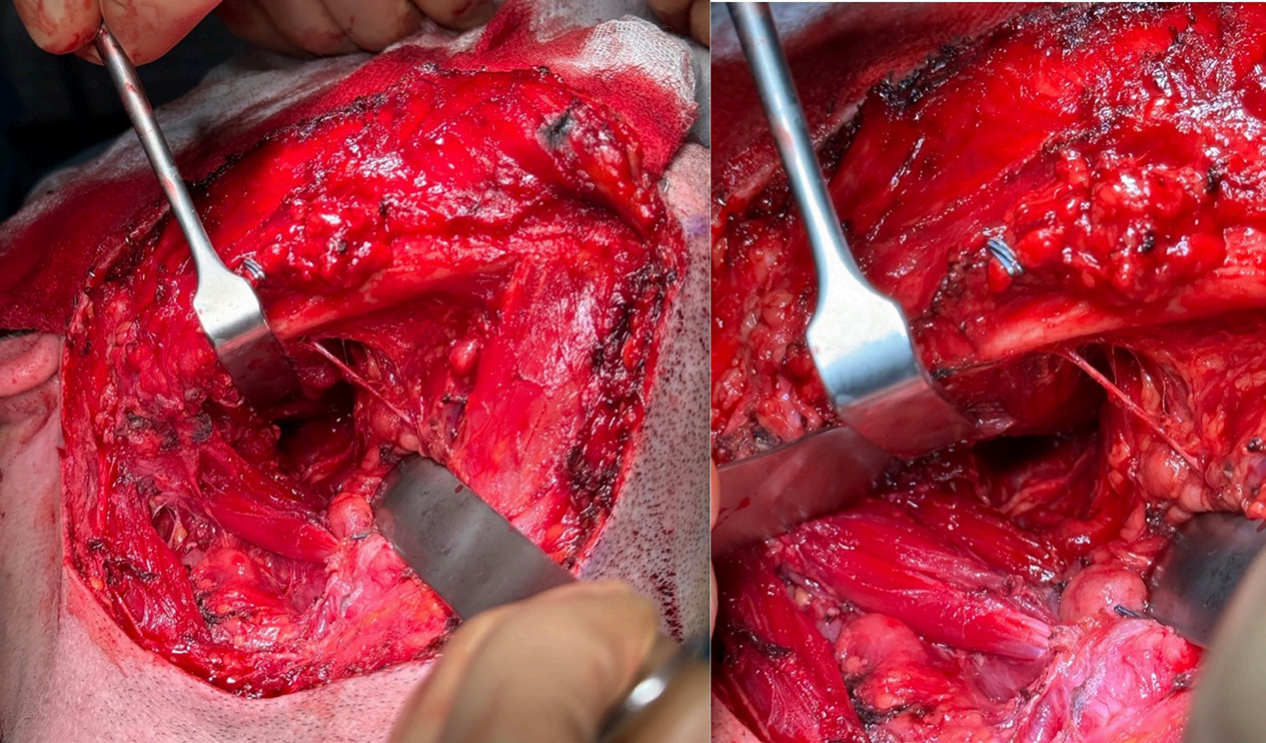
Achieved complete excision without compromising nearby critical anatomical structures.

A transcervical approach was performed to access the right parapharyngeal space. During the procedure, the marginal branch of the facial nerve was visualized and preserved; the facial vessels were individualized and ligated; and then the submaxillary gland was rejected. Proceeding medial to the anterior belly of the digastric, the mass was located. Once the lesion was identified, a blunt dissection was performed respecting its capsule (Fig. 2). Complete excision was performed without damaging nearby relevant anatomical structures (Fig. 3). The tumor measured 6.7 cm in its largest diameter. Histopathology of the surgical specimen confirmed the diagnosis of pleomorphic adenoma. The postoperative period was uneventful and the drain that was placed in the surgical site could be removed the following day.

## Discussion

The incidence of tumors of the parapharyngeal space in the head and neck ranges from 0.5% to 1% (2). Due to the deep position of this space and the slow growth of benign tumors, it is not uncommon for them to reach a large size at the time of diagnosis, as in the clinical case described (3). An added challenge in the approach to the lesion described in the text above was the intimate contact between the lesion and the carotid vascular sheath, making it highly susceptible to damage during excision.

Considering the complexity of the surrounding anatomical structures, surgical approaches to parapharyngeal space are varied. The transcervical approach provides direct access to the middle and lower portions, thus allowing adequate visualization of cranial nerves and vessels. However, it poses limitations in the treatment of malignant tumors located in the upper third of this space, particularly if they are adjacent to the skull base. The combined transcervical-parotid approach is indicated mainly for tumors located in the deep parotid lobe and in intimate contact with the branches of the facial nerve. Mandibulotomy is usually reserved for malignant, recurrent, or large tumors involving or in close contact with the internal carotid and/or the skull base (5). Furthermore, thanks to the development of endoscopy, transoral or transnasal surgery is becoming more and more common in this type of lesion (6,7). The literature shows that there is no unified conclusion on the choice of surgical approach, which mainly depends on the size and location of the tumor, the histology, and the surgeon’s experience (8). In the case of our patient, a cervical approach was performed, since the histology of the tumor indicated it was benign.

The transcervical approach offers direct access to the middle and lower portions of the parapharyngeal space, with good visualization and control of the relevant anatomical structures housed in this space. Furthermore, this approach, besides being optimal for benign lesions, is also associated with less morbidity and a quicker recovery compared to more extensive approaches.

For all these reasons, a transcervical approach was chosen for the procedure. However, it’s important to note that the choice of surgical approach depends on various factors, and the advantages of the transcervical approach should be weighed against the specific characteristics of the tumor, patient factors, and the surgeon’s expertise. For tumors in the upper third of the parapharyngeal space or near the skull base alternative approaches may be explored.

Moreover, preoperative virtual planning of the case using 3D technology provides a better understanding of the patient’s anatomy and a more personalized treatment. Each patient’s anatomy is unique, through 3D virtual planning, strategies can be tailored based on the specific patient characteristics, leading to more precise and individualized interventions.

In addition, the possibility of having available radiological images and virtual planning through navigation software facilitates decision-making during surgery and enhances surgical precision, thus reducing the risk of damage to critical structures and improving postoperative outcomes.

## Data Availability

The datasets used and/or analyzed during the current study are available from the corresponding author.
